# Copper availability controls niche differentiation between comammox *Nitrospira* and ammonia-oxidizing bacteria

**DOI:** 10.1093/ismeco/ycag135

**Published:** 2026-05-17

**Authors:** Kazuyoshi Koike, Garrett J Smith, Nao Okuda, Ryoma Konno, Shizuka Watanabe, Yoshihito Kusunoki, Shuji Kawakami, Theo A van Alen, Maartje A H J van Kessel, Ryoko Yamamoto-Ikemoto, Sebastian Lücker, Norihisa Matsuura

**Affiliations:** Graduate School of Natural Science and Technology, Kanazawa University, Kakuma, Kanazawa 920-1192, Japan; Department of Microbiology, Radboud Institute for Biological and Environmental Sciences, Radboud University, Nijmegen, The Netherlands; Center of Microbiome Science, The Ohio State University, Columbus, OH 43210, United States; Graduate School of Natural Science and Technology, Kanazawa University, Kakuma, Kanazawa 920-1192, Japan; Faculty of Geosciences and Civil Engineering, Kanazawa University, Kakuma, Kanazawa 920-1192, Japan; Faculty of Geosciences and Civil Engineering, Kanazawa University, Kakuma, Kanazawa 920-1192, Japan; Faculty of Geosciences and Civil Engineering, Kanazawa University, Kakuma, Kanazawa 920-1192, Japan; Department of Civil and Environmental Engineering, National Institute of Technology, Nagaoka College, 888, Nishi-Katagai, Nagaoka 940-8531, Japan; Department of Microbiology, Radboud Institute for Biological and Environmental Sciences, Radboud University, Nijmegen, The Netherlands; Department of Microbiology, Radboud Institute for Biological and Environmental Sciences, Radboud University, Nijmegen, The Netherlands; Faculty of Geosciences and Civil Engineering, Kanazawa University, Kakuma, Kanazawa 920-1192, Japan; Department of Microbiology, Radboud Institute for Biological and Environmental Sciences, Radboud University, Nijmegen, The Netherlands; Faculty of Geosciences and Civil Engineering, Kanazawa University, Kakuma, Kanazawa 920-1192, Japan

**Keywords:** nitrification, comammox *Nitrospira*, metagenomics, copper availability, bioreactor, CARD-FISH

## Abstract

The biological oxidation of ammonia, the first step of nitrification, is central to biological water purification processes for nitrogen removal. For drinking water treatment, particularly sourced from groundwater, low concentrations of available copper often limit the efficiency of nitrification. Copper dosing both enhances nitrification and affects the composition of the nitrifying microbial community. The mechanisms underlying the effect of copper on nitrifying community composition, ammonia oxidation, and subsequent nitrogen removal processes remain unknown. The objective of this study was to confirm the effects of copper availability on the relative abundance of complete (comammox) and canonical ammonia-oxidizing bacteria (AOB) in nitrifying communities within the drinking water treatment plant and to determine differences in their copper transport mechanisms. Comparative metagenomic analysis revealed that, unlike most AOB, many comammox *Nitrospira* encode PcoB/CopB-type high-affinity copper uptake systems, indicating that they are more competitive in low-copper environments. This niche adaptation was confirmed in laboratory-scale bioreactors, which showed that comammox *Nitrospira* became dominant under copper-limited conditions, while AOB dominated at high copper concentrations. Furthermore, specific detection of comammox *amoA* mRNA by catalyzed reporter deposition-fluorescent in situ hybridization confirmed that the transcriptional activity of comammox *Nitrospira* was higher compared to AOB under copper limitation. Thus, these results suggest that copper availability may play an important role in shaping the dominant ammonia-oxidizing bacterial guild, with potential implications for engineered water treatment processes.

## Introduction

Efficient ammonium removal during drinking water production is crucial not only because ammonia itself is undesirable in finished drinking water, but also because it promotes the proliferation of heterotrophic and potentially pathogenic bacteria in distribution systems. In addition, residual ammonia supports the growth of ammonia-oxidizing bacteria, which can degrade chloramine [1], a common disinfectant used in drinking water. In the treatment process, ammonium in the raw water is primarily removed through nitrification. This process begins with the oxidation of ammonia by ammonia-oxidizing bacteria (AOB) [2], archaea (AOA) [3], and complete ammonia-oxidizing (comammox) bacteria of the genus *Nitrospira* [4, 5].

Recent studies have shown that comammox bacteria often dominate over AOB and AOA in rapid sand filters, a widely used technology for producing biologically safe drinking water [6–8]. Physiological characterizations revealed that comammox *Nitrospira* have higher ammonia affinities than AOB and many non-marine AOA [9–11]. They also exhibit higher growth yields but lower maximum specific growth rates than AOB and AOA [12]. Consequently, while AOB and AOA may outcompete comammox *Nitrospira* when ammonia is abundant, comammox *Nitrospira* can dominate under certain conditions, such as low dissolved oxygen concentrations [13], long solid retention times [14], or potentially low copper availability, which has been suggested as an additional factor influencing niche differentiation [15].

The role of copper in ammonia oxidation has garnered attention due to its redox-active nature, making it an essential cofactor in several key enzymes [16], including ammonia monooxygenase (AMO). This enzyme catalyzes the oxidation of ammonia to hydroxylamine [17], the first and often rate-limiting step of nitrification. Copper addition has been demonstrated to enhance AMO activity [18], and copper dosing in full-scale drinking water treatment systems has been found to improve ammonium removal [15, 19–21]. We previously reported that comammox *Nitrospira* dominated in a full-scale bioreactor treating groundwater with relatively high ammonium concentrations (9.3 ± 0.5 mg NH_4_^+^-N L^−1^). Ammonium removal was still incomplete after one year of operation, but was improved after the introduction of copper wires into the system, which led to a shift in the dominant comammox *Nitrospira* species within the community and a concurrent increase in the overall dominance of comammox *Nitrospira*. With further copper addition, the dominant nitrifier in the community shifted to AOB affiliated with the *Nitrosomonadaceae* family [15]. Therefore, copper availability is also suggested to play a crucial role in determining the niche of ammonia oxidizers, underscoring the functional diversity and ecological complexity of these organisms.

A frequently used technique to track population changes in mixed microbial communities is fluorescence in situ hybridization (FISH). However, DNA-based methods such as qPCR can only quantify gene abundance and do not provide direct information on the transcriptional activity of microorganisms. In addition, distinguishing between comammox and canonical *Nitrospira* (nitrite-oxidizing bacteria, NOB) using 16S rRNA-targeted approaches is challenging [12]. To overcome these limitations, a method is needed for the specific in situ detection of metabolic functions, such as ammonia oxidation, at the level of active cells. Catalyzed reporter deposition (CARD)-FISH-based detection of mRNA molecules is a powerful tool for detecting transcriptional activity in situ [22]. As comammox *Nitrospira* possess a phylogenetically distinct AMO compared to AOB and AOA [4, 5], it should be feasible to specifically detect them using probes targeting the *amoA* transcripts. However, an mRNA-targeted detection method for nitrifying microorganisms, specifically to distinguish between canonical and comammox *Nitrospira* by targeting the *amoA* mRNA*,* has not been developed.

In this study, we conducted a metagenomic analysis of samples from a full-scale nitrifying groundwater-treatment bioreactor and compared copper-related genes in metagenome-assembled genomes (MAGs) of nitrifying microorganisms. Independently, we furthermore operated continuous laboratory-scale bioreactors to confirm that these genomic differences influence habitat segregation. We monitored changes in the nitrifying bacterial community composition and nitrification performance at varying copper concentrations. Additionally, we developed CARD-FISH oligonucleotide probes targeting the *amoA* mRNA of comammox *Nitrospira* to confirm their identity and transcriptional activity. This comprehensive approach provides insights into the role of copper in shaping the ecological niches of ammonia-oxidizing microorganisms.

## Materials and methods

### Biomass sampling, DNA extraction, and metagenome sequencing

Biomass samples for DNA extraction were collected from a full-scale bioreactor treating ammonium-rich groundwater, as described in our previous study [15]. Briefly, the bioreactor had a capacity of 13.5 m^3^ and contained 5 mm square polyurethane sponge carriers (Technoform Japan, Aich, Japan), occupying 30% of the reactor volume. Biomass was collected from the sponge carriers. No seed sludge was used for inoculation, and groundwater containing 9.3 ± 0.5 mg NH_4_^+^-N L^−1^ was continuously supplied to the bioreactor from an anoxic aquifer located 80 m below ground. The flow rate of groundwater through the bioreactor varied from 1.8 to 14.9 m^3^ h^−1^. The bioreactor was operated continuously for 691 days. On Day 380, circularly wound copper wire immersion was started to supply additional copper. Biomass samples were taken at seven intervals (Days 96, 231, 306, 393, 404, 488, and 602). DNA was extracted from the collected biomass using the FastDNA SPIN Kit for Soil (MP Biomedicals, Santa Ana, CA, USA) according to the manufacturer’s instructions (see Supplemental Methods for details) and sequenced on the Illumina MiSeq (Days 96, 231, 306, 393, 404, and 602) and HiSeq X systems (Days 231, 306, 393, 404, and 488; Illumina, San Diego, California, USA). Additionally, long-read sequencing was conducted on all samples using the Oxford Nanopore MinION or GridION platforms (Flow Cell version R9.4.1; Oxford Nanopore Technologies, Oxford, UK). Base calling was performed using Guppy v4.0.11 with the dna_r9.4.1_450bps_hac.cfg model (Oxford Nanopore Technologies). More detailed information on Illumina and Nanopore library preparation and sequencing is provided in the Supplemental Methods.

### Metagenome assembly, binning, and dereplication

This study employs the recently recommended [23] comprehensive approach for metagenome sequencing, assembly, and analysis, focusing on samples processed with both Illumina and Nanopore sequencing technologies, as described in more detail in the Supplemental Methods. The raw sequencing reads from Illumina (MiSeq and HiSeq) were subjected to rigorous quality control, including trimming, filtering, and adapter removal using BBDuk (BBTools v37.76; https://sourceforge.net/projects/bbmap/). Nanopore reads were filtered for length, and chimeras were removed using BBMap (BBTools v37.76) and Porechop v0.2.4 (https://github.com/rrwick/Porechop). Quality-controlled reads (QC reads) were then hybrid-assembled using either (i) a long-read-first or (ii) a short-read-first approach, depending on the amount of Nanopore data available. For samples sequenced on both MiSeq and HiSeq platforms (days 231, 306, 393, and 404), the datasets were processed separately. Each dataset was independently combined with the corresponding Nanopore reads for hybrid assembly.

(i) In the long-read-first strategy, QC Nanopore reads were assembled de novo using Flye v2.9 [24]. The resulting assemblies were statistically evaluated using BBMap and SeqKit v0.7.1 [25]. Contigs were classified as circular or non-circular via the command line and BBMap. For error correction, QC Nanopore reads were mapped to the assemblies using Minimap2 v2.16-r922 [26] and corrected using Racon v1.3.1 (https://github.com/lbcb-sci/racon). Only non-circular contigs > 4000 bp were used in this step. This error correction was performed iteratively, with the second round using the error-corrected contigs from the first round as references. Subsequently, the circular and corrected non-circular contigs were polished multiple times using QC Illumina reads through mapping with BBMap and polishing using Pilon v1.23 [27]. After each round of short-read polishing, protein-coding genes were predicted by Prodigal v2.6.3 [28], and the “contamination level” (redundancy) of the assembly was assessed using CheckM v1.1.3 [29]. The redundancy reflects the number of duplicated marker genes recovered and will increase when frameshifts typical of Nanopore sequencing are fixed by short-read polishing [23]. The polishing round with the largest apparent contamination was then selected for downstream analysis, which was reached after the seventh polishing round for the non-circular contigs and after the fifth polishing round for the circular contigs.

(ii) For the short-read-first approach, QC Illumina reads were assembled using metaSPAdes v3.14.0 [30], supplemented with QC Nanopore reads to close gaps and resolve repeats. The resulting hybrid assemblies were evaluated using BBMap.

For metagenome binning, all QC read sets were used. The average coverage of contigs from both assembly strategies was estimated using the QC MiSeq, HiSeq, or Nanopore reads using mmlong_readcoverage v0.1.2 (https://github.com/SorenKarst/mmlong). Then, the three sets of read-depth mapping data were combined into a single file using the tidyverse package v1.3.1 [31] in R v3.4.4 [32]. Finally, the coverage information was provided to MetaBAT 2 v2.12.1 using the –abdFile option [33] for the automatic binning of the polished, non-circular contigs.

All metagenome-assembled genomes (MAGs) were dereplicated with dRep v3.5.0 at a strain-level average nucleotide identity (ANI) threshold of 99% (settings –completeness 70, –contamination 10, –cov_thresh 0.5, –S_ani 0.99) [34]. The completeness and contamination were evaluated using CheckM2 v1.0.2 [35]. The presence of 16S, 23S, and 5S rRNA genes was identified using Barrnap v0.9 (https://github.com/tseemann/barrnap), while tRNA counts and types were determined with tRNAscan-SE v2.0.12 [36]. MAGs were classified as medium-quality (>70% completeness, <10% contamination) or high-quality (>90% completeness, <5% contamination, complete rRNA operon, and > 18 tRNAs) based on standard metrics [37], and MAGs with completeness <70% and contamination >10% were excluded. Taxonomic classification was performed using GTDB-Tk v2.4.0 [38] with the Genome Taxonomy Database (GTDB) r220 [39] and functional annotations were carried out using DFAST v1.3.1 with the options –use_trnascan bact –use_prodigal to predict tRNAs and coding sequences (CDS) [40]. Additional assignments to Kyoto Encyclopedia of Genes and Genomes (KEGG) Orthologs (KO) were performed using KofamKOALA v2024-12-02 [41] and mapped to the KEGG database [42] using KEGG-Decoder v1.3 [43]. The relative abundances of nitrifiers were estimated using CoverM v0.6.1 [44] by mapping quality-controlled Illumina reads to the dereplicated MAGs, with relative abundances averaged across MiSeq and HiSeq datasets when both were available.

### Comparative genome analysis

The functional gene content of the nitrifier MAGs obtained in this study was compared to available nitrifier genomes. All available MAGs of the order *Nitrospirales* and the family *Nitrosomonadaceae* on the GTDB r202 (165 and 132 genomes, respectively) were selected as references, and the corresponding assemblies downloaded from the National Center for Biotechnology Information (NCBI) FTP server (https://www.ncbi.nlm.nih.gov/; March 18, 2022) using ncbi-genome-download v0.3.1 (https://github.com/kblin/ncbi-genome-download) with the options –section refseq and –section genbank. The downloaded genomes were dereplicated using dRep v3.5.0 with an estimated completeness >90% and contamination <10% (–completeness 90, –contamination 10, –cov_thresh 0.5), retaining 80 and 45 genomes for the *Nitrospirales* and *Nitrosomonadaceae*, respectively. Gene calling for all genomes was performed using Prodigal v2.6.3 with the -p meta setting. Translated amino acid sequences were mined using hidden Markov models (HMM) [45] available from the Pfam 35.0 database [46] to identify protein-coding genes involved in nitrification and copper transport with hmmlearn v0.2.8 (https://github.com/hmmlearn/hmmlearn) (see Supplementary Table 1 for the HMM models used). To reduce the number of false positive hits for copper transport proteins, the identified genes were extracted and filtered using BLAST [47] against databases downloaded from TrEMBL and Swiss-Prot (https://www.uniprot.org/, 9 March 2025) and created with makeblastdb, applying a cut-off setting (−evalue 1e-5, −qcov_hsp_perc 50) and manual filtering at 30% amino acid identity. Nitrification marker proteins (AmoA, Hao, NxrA) were also confirmed by BLAST (−evalue 1e-6, −qcov_hsp_perc 70) against in-house databases downloaded from the TrEMBL and Swiss-Prot (11 November 2022) and manually filtered at 45% identity. Finally, rRNA genes were predicted and 16S rRNA genes were extracted using Barrnap v0.9.

### Phylogenomic and phylogenetic analyses

For phylogenomic analysis, the up-to-date bacterial core gene pipeline v3.0 [48] was used with default parameters to extract, align, and concatenate phylogenetic marker gene sets from all nitrifier MAGs and reference genomes. For phylogenetic analysis of single marker genes, reference sequences were obtained from NCBI (18 March 2022) and aligned with the MAG gene sequences using MAFFT v7.215 [49]. 16S rRNA genes were additionally filtered at >1000 bp using SeqKit. Phylogenomic and 16S rRNA gene, AmoA, and Hao-based phylogenetic trees were calculated using IQ-TREE v2.1.3 [50] with the best-fit model (−m MFP) and 1000 ultrafast bootstrapping replications (-B 1000). The phylogenomic tree was based on concatenated amino acid sequences of core gene sets. The 16S rRNA gene phylogeny was based on nucleotide sequences, whereas AmoA and Hao phylogenies were inferred from amino acid sequences. Final trees were visualized in iTOL v6.6 [51].

### Laboratory-scale bioreactor operation and physicochemical characterization

**Figure 1:**
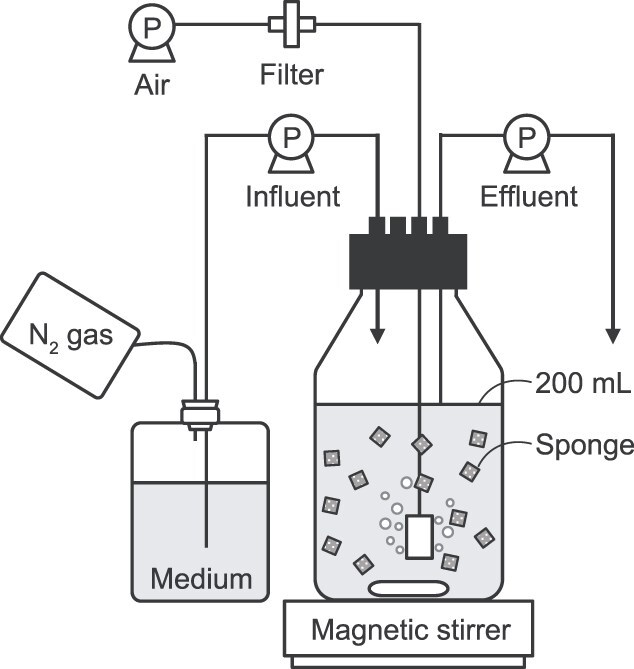
Schematic illustration of the laboratory-scale bioreactors. Two parallel reactors with low and high dissolved copper concentrations (0.2 and 200 μg L^−1^, respectively) were operated for 180 days. Medium samples for analysis were taken from the effluent vent.

To study the impact of varying copper concentrations on ammonia-oxidizing microorganisms, two 250 ml continuous bioreactors were used, each with a working volume of 200 ml (Fig. 1). They received mineral medium (see Supplemental Methods) containing 44.0 mg L^−1^ (NH_4_)_2_SO_4_ and either 0.54 μg L^−1^ or 537 μg L^−1^ CuCl_2_ × 2H_2_O for the low dissolved copper (dCu) or high-dCu condition, respectively, corresponding to 0.2 μg L^−1^ or 200 μg L^−1^ of dCu, respectively. The low-dCu concentration was comparable to the groundwater used in the full-scale bioreactor used as biomass source [15], and the high-dCu concentration was 1000-fold higher. The medium was adjusted to pH 7.8 with 1 M KHCO_3_ and purged with nitrogen gas to reduce oxygen levels to prevent ammonia oxidation in the storage tank.

The bioreactors were inoculated with 199 ml of media and eight biomass-containing sponges (1 ml) collected in November 2021 from the full-scale groundwater treatment bioreactor [15], and supplemented with 160 fresh 5 mm square sponge carriers (Techno Foam Japan, Anjo, Japan; equivalent to 10% of the working volume). The bioreactors were stirred at 150 rpm, maintained at 25°C in the dark, and continuously aerated with ambient air (1 L min^−1^). The culture medium was continuously fed to the bioreactor at a flow rate of 100 ml h^−1^ for 180 days. Effluent samples were collected and filtered using a 0.2 μm pore size hydrophilic polytetrafluoroethylene (PTFE) membrane (Merck KGaA, Darmstadt, Germany). Concentrations of ammonium, nitrite, and nitrate ions were determined via ion chromatography (IC) on a Shimadzu IC system (Prominence HIC-NS/HIC-SP, Shimadzu, Kyoto, Japan) equipped with columns for cation and anion determination (Shim-pack IC-C4 and IC-SA2, Shimadzu, respectively) and an electroconductivity detector (CDD-10A SP, Shimadzu).

### 16S rRNA gene amplicon sequencing and data processing

To study the dynamics within the nitrifying microbial communities, sponges were collected from each bioreactor at 0 (inoculum sponges from a full-scale bioreactor), 15, 35, 50, 70, 90, 120, 150, and 180 days. Biomass was extracted by squeezing the sponges. The detailed protocols for sample collection, amplicon sequencing, and data processing are described in the Supplemental Methods. Briefly, the PCR was conducted using the primers 515F and 806R [52], followed by library preparation and sequencing on an Illumina MiSeq platform. The raw sequencing data underwent initial trimming and filtering using BBDuk, followed by primer trimming with Cutadapt v4.1 [53]. Subsequent steps involved length trimming, quality filtering, error rate learning, read dereplication, Amplicon Sequence Variant (ASV) inference, paired read merging, and chimera removal using DADA2 v1.30.0 [54]. Finally, taxonomic classification of ASVs was performed using the SILVA 138.1 prokaryotic SSU taxonomic training data formatted for DADA2 [55].

### Comammox *amoA* gene-specific probe design

To specifically detect comammox *Nitrospira* in the laboratory-scale bioreactors, a probe targeting comammox *amoA* mRNA was developed. The detailed procedure for probe design is described in the Supplemental Methods. Three probes were finally selected: Ntsp_amoA_003 (5' − ATTTCATCGGTTCTAAAC−3′), Ntsp_amoA_642 (5' − GAGATCATGGTGCTGTGA−3′), and Ntsp_amoA_249 (5' − GCTGACGATAGTTCACCCAA−3′). The number of mismatches between each probe and the curated *amoA* reference sequences within the family *Nitrospiraceae* was assessed in silico (Supplementary Table 2). All three probes were synthesized (Japan Bio Services, Saitama, Japan) with a horseradish peroxidase (HRP) modification at the 5′ end.

### CARD-FISH-based detection of comammox *Nitrospira*

To ensure the accuracy of the in situ hybridization and fluorescence-based detection, all materials and equipment used were RNase-free. Biomass samples were collected on Day 180, as previously described, and an equal volume of 1× PBS was added. The samples were fixed with 12% paraformaldehyde (PFA) in a 2:1 ratio (vol/vol), resulting in a final concentration of 4% PFA, and incubated at 4°C for 2 h. After fixation, the samples were washed three times with 1× PBS, centrifuged for 10 min (8000 × g), and stored in PBS/ethanol (1:1) at −20°C until further use.

CARD-FISH was performed on duplicate samples using a modified protocol [22, 56] (see Supplemental Methods for details). Briefly, fixed samples were embedded in low-melting-point agarose, treated with lysozyme, dehydrated in ethanol, and hybridized with HRP-labeled probes. After washing, tyramide signal amplification of Cy3 was performed, and samples were counterstained with DAPI before fluorescence microscopy. Negative controls included *Escherichia coli* DH5α and biomass samples without probes. Images were captured using a 100× oil immersion objective on an ECLIPSE Ni-U microscope (Nikon, Tokyo, Japan). All experiments were conducted in technical duplicates.

## Results and discussion

### Recovery of high-quality nitrifier MAGs through hybrid metagenomic assembly

In our previous study, during the ~700 days of operation of a full-scale groundwater treatment reactor under copper-limited conditions, we observed that comammox *Nitrospira* were the dominant ammonia oxidizers. Notably, moderate copper addition enhanced nitrification efficiency and reinforced the dominance of comammox *Nitrospira*, whereas excessive copper dosing led to a shift toward *Nitrosomonas*-dominated ammonia oxidation [15].

In this study, metagenomic sequencing was performed at seven time points to examine why shifts in the nitrifying community occurred in the full-scale groundwater treatment reactor. Draft metagenome bins were recovered through hybrid assembly strategies using differential coverage binning. Both long-read-first and short-read-first assembly are widely used methods, but the choice of the starting read set depends on the richness of each data set [57]. For time points with limited long-read data (<2 Gbp in total or N50 < 1 Kbp, which applied to Days 231, 488, and 602), long-read-first assemblies produced few high-quality contigs and bins (Supplementary Tables 3 and 4), and switching to short-read-first assemblies improved bin recovery. For samples with both MiSeq and HiSeq data, the datasets were independently processed and used for hybrid assembly. The resulting assemblies were independently binned, and the higher-quality bins were selected for further analyses. Overall, strain-level dereplication of the bins yielded 67 medium-quality or better MAGs, of which 30 originated from long-read-first and 37 originated from short-read-first hybrid assemblies. Notably, long-read-first assemblies produced six high-quality single-contig MAGs, three of which were circular genomes (>98% completeness, <1% contamination). Thus, high-quality MAGs were primarily generated through long-read-first assembly, with subsequent polishing using short reads. The integration of contigs from different assembly methods, combined with final MAG dereplication, proved effective for constructing higher-quality genomes, as suggested recently [23].

12 MAGs were taxonomically classified as nitrifying genera (7 *Nitrospira* and 5 *Nitrosomonas* MAGs; Table 1, Supplementary Table 5). The ammonia oxidation pathway was conserved in all *Nitrosomonas* MAGs except SFBR_MAG_46. The complete ammonia oxidation pathway was identified in two *Nitrospira* MAGs, SFBR_MAG_26 and SFBR_MAG_41, whereas the remaining *Nitrospira* MAGs harbored the nitrite oxidation pathway, with the exception of SFBR_MAG_52 (Supplementary Fig. 1). The absence of these key functional genes in SFBR_MAG_46 and SFBR_MAG_52 may be due to insufficient sequencing depth or assembly-related limitations. These results indicate that SFBR_MAG_26 and SFBR_MAG_41 represent comammox *Nitrospira*. To further examine the temporal dynamics of nitrifiers, quality-controlled Illumina reads were mapped to the nitrifier MAGs to estimate their relative abundances over time. Based on this analysis, comammox *Nitrospira* reached their highest relative abundance on day 404 (40.8%), whereas AOB were most abundant on day 602 (9.6%) (Table 1). These MAG-based abundance patterns are consistent with our previous observations based on 16S rRNA gene amplicon sequencing from the same full-scale reactor [15]. As expected, all *Nitrosomonas* MAGs encoded the Calvin-Benson-Bassham (CBB) cycle for carbon fixation, whereas the reductive citric acid cycle (rTCA) was conserved in the *Nitrospira* species (Supplementary Fig. 1). It is well established that *Nitrospira* species, including comammox *Nitrospira*, utilize the rTCA cycle for carbon fixation, whereas AOB rely on the CBB cycle [12]. Consistent with this, the comammox *Nitrospira* identified in this study also encoded the rTCA cycle. Given that the rTCA cycle is more energy-efficient than the CBB cycle [12], this observation is consistent with an adaptation of comammox *Nitrospira* to oligotrophic environments.

**Table 1:** Characteristics of dereplicated nitrifier MAGs obtained in this study.

**MAG ID**	**Hybrid assembly type**	**Read combination**	**Species (GTDB-R220)**	**Nitrifier guilds**	**Completeness (%)**	**Contamination (%)**	**N50 (bp)**	**Size (Mbp)**	**GC content**	**CDS**	**Number of contigs**	**Circular contig**	**5S rRNA**	**16S rRNA**	**23S rRNA**	**tRNAs**	**MIMAG quality**	**Mean relative abundance (%)**						
																		**Day 96**	**Day 231**	**Day 306**	**Day 393**	**Day 404**	**Day 488**	**Day 602**
SFBR_MAG_06	Long-read-first	Nanopore - > MiSeq	Unclassified Nitrospira_D	NOB	86.2	1.0	9,92 798	3.4	0.58	3346	5	−	1	1	1	41	Medium	7.17	4.82	2.80	3.49	3.84	3.23	3.33
SFBR_MAG_26	Long-read-first	Nanopore - > HiSeq	Unclassified Nitrospira_D	Comammox	100	1.3	34,99 053	3.7	0.56	3670	3	−	1	1	1	47	High	0.91	6.30	9.05	4.65	3.84	1.01	0.24
SFBR_MAG_28	Long-read-first	Nanopore - > HiSeq	Unclassified Nitrospira_D	NOB	100	0.1	35,86 525	3.6	0.58	3519	1	−	1	2	2	48	High	5.50	8.52	4.54	5.74	6.56	3.98	2.77
SFBR_MAG_29	Long-read-first	Nanopore - > HiSeq	Nitrospira_A sp900170025	NOB	100	1.1	36,42 607	4.3	0.59	4143	3	−	1	1	1	46	High	0.87	0.25	2.13	2.53	2.03	7.56	5.06
SFBR_MAG_40	Long-read-first	Nanopore - > MiSeq	Unclassified Nitrospira_D	NOB	86.6	0.1	6,18 013	3.5	0.58	3898	6	−	1	0	0	41	Medium	6.05	7.86	4.42	5.90	6.82	4.24	2.86
SFBR_MAG_41	Long-read-first	Nanopore - > HiSeq	Unclassified Nitrospira_D	Comammox	99.9	0.7	46,05269	4.6	0.54	4404	1	+	1	1	1	46	High	18.87	5.34	11.70	29.02	36.98	18.75	3.76
SFBR_MAG_52	Short-read-first	HiSeq - > Nanopore	Unclassified Nitrospira_D	NOB	83.5	0.7	35 354	3.4	0.58	3417	156	−	0	0	0	22	Medium	6.68	4.47	2.58	3.23	3.54	3.28	3.60
SFBR_MAG_27	Long-read-first	Nanopore - > HiSeq	Unclassified Nitrosomonas	AOB	91.4	1.2	61 338	3.1	0.47	3455	74	−	0	0	1	39	Medium	0.59	0.21	1.23	1.35	0.53	0.46	1.20
SFBR_MAG_46	Short-read-first	HiSeq - > Nanopore	Unclassified Nitrosomonas	AOB	76.7	0.8	5656	2.1	0.48	2248	418	−	0	0	0	21	Medium	0.43	0.14	0.89	1.01	0.39	0.35	0.89
SFBR_MAG_61	Short-read-first	MiSeq - > Nanopore	Unclassified Nitrosomonas	AOB	100	1.8	1,39 520	2.8	0.42	2502	25	−	0	0	0	37	Medium	NA	0.02	NA	NA	0.02	0.04	4.24
SFBR_MAG_64	Short-read-first	MiSeq - > Nanopore	Unclassified Nitrosomonas	AOB	88.4	1.0	15 672	2.6	0.48	2464	249	−	0	0	0	16	Medium	0.51	0.17	1.08	1.19	0.46	0.42	1.06
SFBR_MAG_65	Short-read-first	MiSeq - > Nanopore	Unclassified Nitrosomonas	AOB	100	0.0	60 785	3.2	0.45	2958	95	−	0	0	0	30	Medium	0.17	0.11	0.16	0.14	0.05	0.03	2.25

### Genome-centric analysis of two distinct comammox *Nitrospira* in a full-scale bioreactor

**Figure 2:**
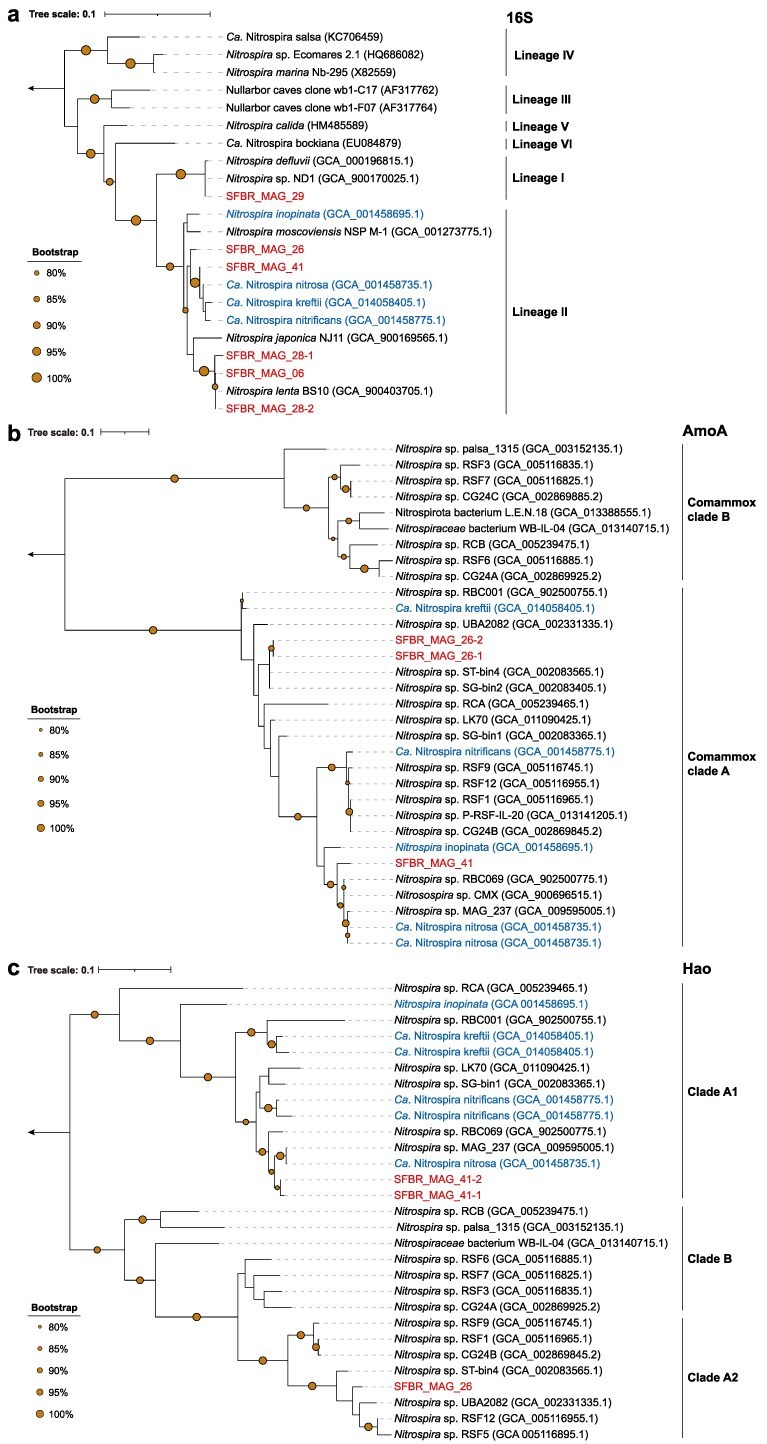
16S rRNA gene, AmoA, and HaoA-based phylogenetic diversity analysis of *Nitrospira* MAGs recovered in this study. (a) 16S rRNA gene-based maximum-likelihood (ML) tree constructed from nucleotide sequences. The arrow indicates the position of the outgroup, which consisted of two *Leptospirillum* species (GCA_000284315.1 and GCA_000299235.1). (b) AmoA-based ML tree constructed from amino acid sequences. Sequences from three *Nitrosomonadaceae* genome assemblies (GCA_003050865.1, GCA_900167395.1, and GCA_003051105.1) were used to root the tree. (c) Hao-based ML tree constructed from amino acid sequences, rooted with three *Nitrosomonadaceae* sequences (extracted from GCA_003050865.1, GCA_900167395.1, and GCA_003051105.1). Genes obtained in this study are shown in red, cultured comammox *Nitrospira* in blue, and reference sequences in black. The brown circles indicate statistical branch support from 1000 ultrafast bootstrapping replications. The scale bars represent 10% sequence divergence.

Phylogenetic classification of the 16S rRNA gene sequences extracted from five of the seven *Nitrospira*-affiliated MAGs (Fig. 2a) showed that SFBR_MAG_29 clusters with *Nitrospira* sublineage I, whereas all the other MAGs belong to sublineage II. In the latter, two sequences were highly similar to each other and most closely related to the canonical nitrite oxidizer *N. lenta*, while the remaining two MAGs appear to represent comammox *Nitrospira* (SFBR_MAG_41 and SFBR_MAG_26), with their 16S rRNA gene sequences being 99.6% and 98.4% identical to *Candidatus* Nitrospira nitrosa, respectively. Confirming their comammox lifestyle, these two MAGs also contained *amoA* genes that belong to the comammox *Nitrospira* clade A (Fig. 2b). The AmoA from SFBR_MAG_41 was placed in the same cluster as *Ca.* N. nitrosa. SFBR_MAG_26 encodes two identical AmoA copies that formed a cluster with *Nitrospira* sp. UBA2082, *Nitrospira* sp. ST-bin4, and *Nitrospira* sp. SG-bin2. According to the phylogeny of hydroxylamine dehydrogenase (Hao), SFBR_MAG_41 and SFBR_MAG_26 are affiliated with comammox clades A1 and A2, respectively (Fig. 2c) [58]. Similar to most genomes in these clades (Fig. 2c), the clade A1 SFBR_MAG_41 possessed two copies of the *hao* gene, whereas the clade A2 SFBR_MAG_26 had only one copy. The divergence between clades A1 and A2 is particularly evident in the Hao phylogeny, where clade A2 Hao sequences cluster more closely with comammox clade B than with clade A1. This pattern has been proposed to have originated through horizontal gene transfer and likely causes ecological divergence among comammox *Nitrospira* lineages [58]. These differences in AmoA and Hao distributions suggest that the two comammox *Nitrospira* within the bioreactor occupy distinct ecological niches and perform different roles within the system despite their overall physiological similarity.

**Figure 3:**
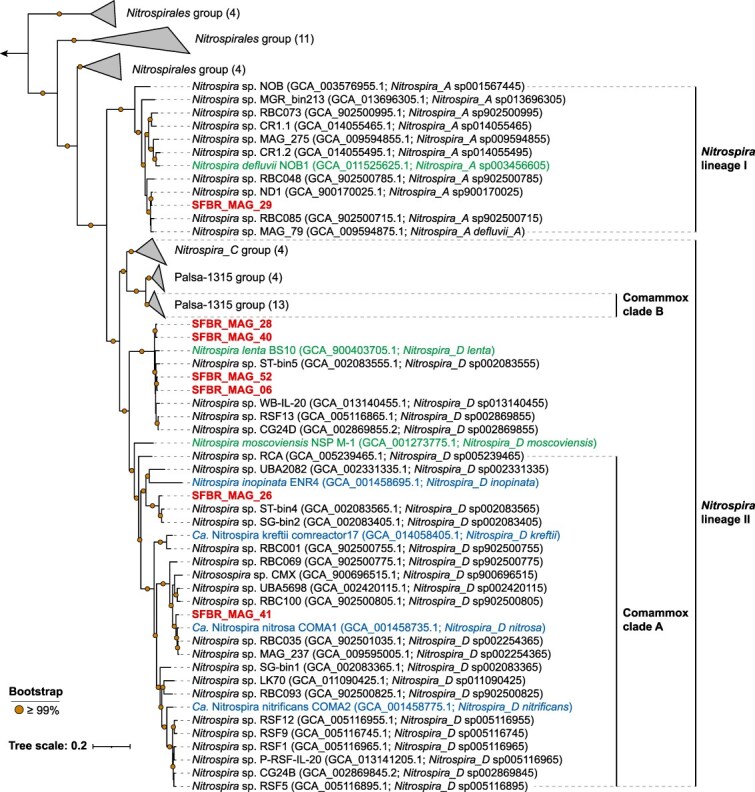
Phylogenomic analysis of the *Nitrospira* MAGs recovered in this study. A maximum-likelihood tree was constructed based on concatenated amino acid sequences of 92 bacterial single-copy core genes. MAGs obtained from the full-scale bioreactor metagenomes are shown in red, cultured comammox and canonical *Nitrospira* in blue and green, respectively, and all other reference sequences in black. The phylogenomic tree was rooted using eight *Leptospirillum* genomes as the outgroup (GCA_000205145.2, GCA_000284315.1, GCA_000299235.1, GCA_000695975.1, GCA_000755505.1, GCA_001186405.1, GCA_001280545.1, and GCA_002002505.1). The outgroup sequences are not shown in the figure for clarity, and their position is indicated by the arrow. Reference genomes are annotated with their NCBI organism name, genome accession number, and GTDB (R220) species name. Brown circles indicate statistical branch support from 1000 ultrafast bootstrapping replications. The scale bar represents 20% sequence divergence.

Phylogenomic analysis further supported three marker gene-based observations (Fig. 3). SFBR_MAG_29 was affiliated with *Nitrospira* sublineage I, while the other six MAGs clustered within *Nitrospira* sublineage II. While four of these sublineage II MAGs were related to *N. lenta*, the two remaining MAGs (SFBR_MAG_41 and SFBR_MAG_26) fell within the comammox clade A. According to their average nucleotide identity (ANI) values, SFBR_MAG_41 is most similar to *Ca.* N. nitrosa at 91.2% identity, while SFBR_MAG_26 has ANI values of 90.6% and 90.5% to the closely related tap water-derived *Nitrospira* sp. SG-bin2 and ST-bin4, respectively. Thus, both MAGs represent novel clade A comammox species.

Importantly, the recovery of full-length 16S rRNA gene sequences from both comammox MAGs allowed a more robust placement of these organisms within *Nitrospira* sublineage II. While comammox *Nitrospira* do not form one monophyletic cluster within *Nitrospira* sublineage II, this study contributes to resolving the 16S rRNA gene-based phylogenetic positioning of comammox *Nitrospira*. Nevertheless, neither the 16S rRNA gene nor the AmoA phylogeny provides sufficient resolution to clearly distinguish between clades A1 and A2. In contrast, consistent with a previous [58], the Hao-based phylogeny in this study separated these clades, suggesting an evolutionary distinction that may underlie subtle differences in ecological adaptation. Although both clades generally share an ecological niche, characterized by complete ammonia oxidation at low ammonium levels, the conserved divergence in Hao indicates potential specialization to distinct sub-niches, which warrants further investigation.

### Copper transport systems among ammonia-oxidizing guilds and their potential role in niche differentiation

**Figure 4:**
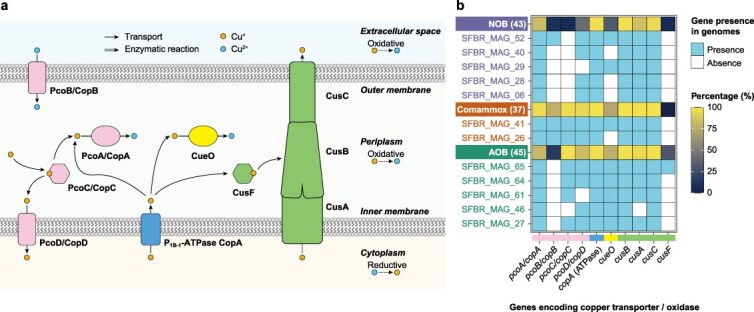
Comparison of copper homeostasis systems encoded in nitrifying bacterial genomes. (a) Schematic diagram of the presumed copper transport pathways in gram-negative bacteria. Proteins with copper transport function are shown as rounded rectangles, periplasmic copper-binding proteins as hexagons, and copper oxidases as ovals. Cu^+^ and cu^2+^ ions are indicated by orange and cyan circles, respectively. Copper transport and enzymatic reduction pathways are indicated by single-line and double-line arrows, respectively. The PcoABCD/CopABCD copper homeostasis systems are shown in pink, the P_1B-1_-type ATPase CopA in blue, the copper oxidase CueO in yellow, and the CusCFBA copper export systems in green. PcoA/CopA, copper oxidase; PcoB/CopB, outer membrane copper import protein (high Cu^2+^ affinity); PcoC/CopC, periplasmic copper binding protein; PcoD/CopD, inner membrane copper transport protein; P_1B-1_-ATPase CopA, Cu^+^ transporting P_1B-1_-type ATPase; CueO, copper oxidase; CusCBA, copper export complex; CusF, periplasmic copper binding protein. (b) Heat map showing the distribution of genes for the copper transporters and oxidases in nitrifying bacteria, with NOB indicated in green, comammox *Nitrospira* in red, and AOB in blue. Gene presence in MAGs from this study is indicated in light blue, their relative abundance in reference genomes is represented by a color gradient. The total number of reference genomes used for comparison is given in parentheses. The multicopper oxidase contains three distinct domains (PF00394, PF07731, PF07732), and each domain was searched for within the putative *pcoA/copA* genes. If no hit was obtained for any domain, the gene was considered absent.

We conducted a comparative genome analysis of comammox *Nitrospira* MAGs obtained in this study together with representative genomes from AOB and canonical *Nitrospira* to investigate the presence and distribution of the copper homeostasis proteins. Copper is an essential yet potentially toxic trace metal at elevated concentrations, and most bacteria employ specific mechanisms to manage intracellular copper concentrations. Many Gram-negative bacteria synthesize copper-transporting P-type ATPases, multi-component copper efflux systems belonging to the resistant-nodule-cell division (RND) family, and multicopper oxidases (MCOs) [59]. Representative components of these systems include CopA, a P_1B-1_ subfamily ATPase within the P-type ATPases, which mediates Cu^+^ efflux from the cytoplasm to the periplasm; the RND system CusCFBA, which exports Cu^+^ from the cytoplasm and periplasm to the outer membrane; and the MCO CueO, which oxidizes periplasmic Cu^+^ to Cu^2+^ (Fig. 4a) [59]. Furthermore, some bacteria encode homologs of the functionally similar PcoABCD or CopABCD copper management systems. The PcoA/CopA encodes an MCO that can functionally substitute for CueO [60], PcoB/CopB mediates Cu^2+^ uptake from the extracellular space to the periplasm [61], and PcoCD/CopCD is responsible for copper uptake from the periplasm into the cytoplasm [62]. The genomic localization of the genes encoding CopCD between the *amoCAB* and *amoED1D2* operons in *Ca.* N. nitrosa and *Ca.* N. nitrificans supports their role in copper uptake [5].

We investigated whether the genomic potential for intracellular copper management differs among nitrifiers. Comparison of the MAGs obtained in this study showed differences in the presence of the PcoABCD/CopABCD system (Fig. 4a). Most of the obtained nitrifier MAGs encoded PcoA/CopA, but PcoB/CopB was characteristic for comammox *Nitrospira* (Fig. 4b). In addition, most AOB and both comammox *Nitrospira* MAGs encoded PcoCD/CopCD, whereas the NOB MAGs lacked PcoC/CopC and, in one case, PcoD/CopD as well (Fig. 4b). Although canonical NOB do not possess AMO, copper may still play roles in other cellular processes, such as electron transport, and its demand is likely lower compared to ammonia-oxidizing microorganisms. Similarly, among the analyzed reference genomes, the presence or absence of *pcoB/copB* gene showed a distinct distribution pattern between the three nitrifier guilds and was almost exclusively found in comammox *Nitrospira* (Fig. 4b). Other copper management systems showed no major differences between AOB and comammox and canonical *Nitrospira*. Almost all nitrifiers encoded the P_1B-1_-type ATPase CopA and the RND superfamily transporter CusCBA, while 32.2%–77.0% encoded the MCO CueO, and 0–30.8% the metallochaperone CusF (Fig. 4b). Thus, the presence of PcoB/CopB almost exclusively in comammox *Nitrospira* suggests they may have enhanced copper-importing capabilities. PcoB/CopB has been proposed to function as a high-affinity copper uptake system [61], which may be especially advantageous under copper-limited conditions. Furthermore, comammox *Nitrospira* are richer in copper-transporting systems than both AOB and canonical *Nitrospira*. Such distinct copper management strategies might contribute to the competitive advantage of comammox *Nitrospira*, particularly in copper-limited environments, despite the higher maximum specific growth rates of AOB [12, 15]. Efficient uptake and management of intracellular copper is crucial for activating the copper centers of the AMO complex, especially given its location within the cytoplasmic membrane and, in the case of AOB, the intracytoplasmic membrane stacks [17]. However, how these reported copper-importing systems contribute to the apparent high copper affinity of comammox *Nitrospira* remains to be experimentally tested.

### Differential responses of nitrifiers in the two identical laboratory-scale bioreactors

**Figure 5:**
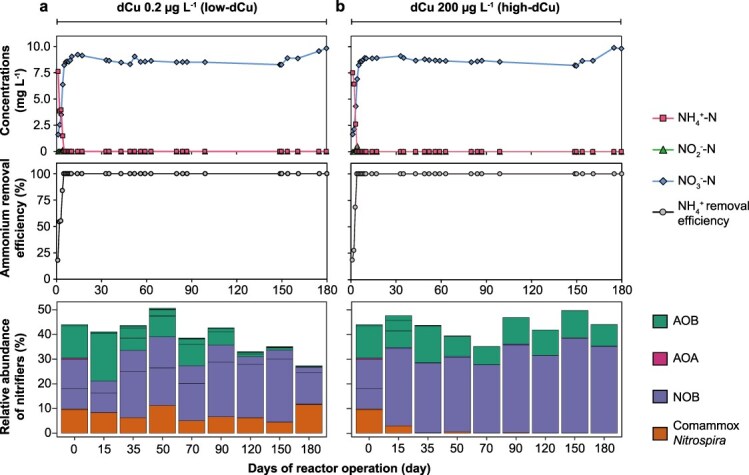
Laboratory-scale bioreactor performance and nitrifier dynamics. Ammonium, nitrite, and nitrate concentrations in the effluent (top), ammonium removal efficiency (middle), and 16S rRNA gene amplicon sequencing-based relative abundance of nitrifiers during the operational period (bottom) in the low-dCu (a) and high-dCu (b) bioreactors are indicated.

To assess whether comammox *Nitrospira* are competitively favored over AOB under copper-limited conditions, two identical laboratory-scale bioreactors were inoculated with biomass from a nitrifying full-scale groundwater treatment bioreactor [15] and operated under copper-limited (low-dCu; 0.2 μg L^−1^) and copper-replete (high-dCu; 200 μg L^−1^) conditions. Complete ammonium removal was achieved within five days in the low-dCu bioreactor (Fig. 5a) and 4 days in the high-dCu bioreactor (Fig. 5b). Both systems maintained similar nitrification performance throughout the operational period (Fig. 5), with the pH kept at 7.5 ± 0.5 and dissolved oxygen maintained at 6.7 ± 0.6 mg L^−1^. Biomass was collected at nine time points for 16S rRNA gene amplicon analysis to assess the nitrifier community composition. By using the 16S rRNA gene sequences retrieved from the *Nitrospira* MAGs obtained from the full-scale drinking water treatment reactor as reference, we could distinguish comammox and canonical *Nitrospira*. Additionally, phylogenetic analysis of 16S rRNA gene amplicons was performed (Supplementary Fig. 2).

The initial inoculum contained a relative abundance of 13.6% AOB, 0.5% AOA, 20.1% NOB, and 9.8% comammox *Nitrospira* (Fig. 5). Over 180 days of operation, canonical *Nitrospira*, which function as nitrite-oxidizing bacteria (NOB), maintained average relative abundances of 23.1% and 31.1% in the low-dCu and high-dCu bioreactors, respectively, with a notable increase in the high-copper condition (Fig. 5). Notably, canonical *Nitrospira* were the only NOB detected in both bioreactors, and no other nitrite-oxidizing genera were observed throughout the operation. In both reactors, the relative abundance of canonical *Nitrospira* substantially exceeded that of ammonia oxidizers, resulting in a pronounced imbalance between ammonia oxidizers and NOB. Such an uneven distribution between ammonia oxidizers and nitrite oxidizers has been frequently reported in engineered nitrifying systems, including drinking water treatment and wastewater treatment processes [21, 63].

The relative abundance of comammox *Nitrospira* in the low-dCu bioreactor varied between 4.5% and 11.8% (Fig. 5a), but rapidly declined in the high-dCu bioreactor after Day 15 and fell below the detection limit by day 180 (<0.007%; Fig. 5b). Contrastingly, the AOB population in the low-dCu bioreactor decreased to 0.6% by Day 180, while they constituted between 7.4% and 15.1% in the high-dCu system (Fig. 5). Consistent patterns were also observed in preliminary laboratory-scale bioreactor experiments, in which comammox *Nitrospira* became dominant under low-dCu conditions, whereas AOB predominated under high-dCu conditions. These preliminary experiments included two independent low-dCu runs using different inoculum and flow rate, as well as one comparative low-dCu versus high-dCu experiment. Detailed methodologies and results of these experiments are provided in the Supplementary Information (Supplementary Fig. 3). The model AOB *Nitrosomonas europaea* grows at copper concentrations ranging from 5.0 to 30.0 μg L^−1^ (78.7 to 472.1 nmol L^−1^) [64], and the marine AOB *Nitrosococcus oceani* strain C-107 has a maximum specific growth rate at a dCu concentration of 2.5–4.9 nmol L^−1^ (0.2–0.3 μg L^−1^) [65]. To date, the copper requirements for optimal growth in comammox *Nitrospira* have not been tested, but a dCu concentration of 20 μg L^−1^ (117.3 nmol L^−1^) was used to cultivate *Nitrospira inopinata* [4]. Here, we observed that in our laboratory-scale bioreactor, comammox *Nitrospira* predominates over *Nitrosomonas*-like AOB at a dCu level as low as 0.2 μg L^−1^. Together, these laboratory-scale bioreactor results are consistent with the possibility suggested by the comparative genomic analysis that distinct copper management strategies may contribute to a competitive advantage of comammox *Nitrospira* under copper-limited conditions. Furthermore, the results observed in our laboratory-scale bioreactors suggest that copper availability is an important ecological factor shaping competition between comammox *Nitrospira* and AOB in nitrifying communities.

Besides copper availability, ammonium concentration is known to influence niche differentiation among ammonia-oxidizing microorganisms and may also have influenced the observed community dynamics in the laboratory-scale bioreactors. As noted above, AOB are generally characterized by higher maximum specific growth rates than comammox *Nitrospira* [12]. Under the high-dCu condition, this physiological characteristic likely enabled AOB to grow rapidly, resulting in a relatively fast decline in the relative abundance of comammox *Nitrospira*. In contrast, under low-dCu conditions, AOB exhibited limited growth, whereas comammox *Nitrospira* increased more gradually over time, likely reflecting their lower maximum specific growth rates. This asymmetry in growth dynamics may explain why AOB did not disappear as rapidly in the low-dCu reactor as comammox *Nitrospira* did in the high-dCu treatment. A recent study conducted under low ammonium concentrations (<1 mg L^−1^) demonstrated that copper dosing increased the relative abundance of AOB, suggesting that copper availability may contribute to shaping nitrifier community structure even under ammonium-limited conditions [21]. Taken together, these observations indicate that the competitive outcomes between AOB and comammox *Nitrospira* may be influenced by copper availability across a range of ammonium concentrations. However, bioreactor-based studies addressing the combined effects of ammonium concentration and copper availability are still limited, and additional experimental work will be necessary to better understand how these two niche-determining factors interact to shape nitrifying communities.

Throughout the operation of the laboratory-scale bioreactors, the only AOA detected was *Ca.* Nitrosotenuis (a non-marine AOA), which was present only in the initial inoculum. Regarding the role of copper in AOA, it has shown that marine AOA exhibit a higher effective copper affinity than marine AOB, even though their genomes encode fewer known copper management protein compared to AOB [65]. The marine AOA *Nitrosopumilus maritimus* strain SCM1 shows maximal growth at a dCu concentration of 70 nmol L^−1^ (4.4 μg L^−1^) [65], it was capable of maintaining growth at lower cupric ion (Cu^2+^) concentrations than *N. oceani* strain C-107 [65, 66]. In addition, non-marine AOA generally have higher maximum specific growth rates than comammox *Nitrospira* [12], and *Ca.* Nitrosotenuis has been reported to exhibit an ammonia affinity comparable to that of comammox *Nitrospira* [9]. This knowledge suggests that non-marine AOA could potentially grow under the low-dCu conditions applied in this study. However, despite this potential, non-marine AOA were not detected in the low-dCu reactor during the operation. Because there is no knowledge on the copper affinity of non-marine AOA, it is difficult to discuss the reason for the loss of competition from the perspective of copper concentration. Instead, other environmental factors (eg, oxygen concentration) are likely involved. For example, AOA are known to exhibit a higher affinity for oxygen than AOB and often display greater competitiveness under low dissolved oxygen conditions [67–69]. Because the reactors in this study were operated under fully oxic conditions, such conditions may have favored AOB and comammox *Nitrospira* over AOA.

The consistent results obtained from the comparative genomic analysis and the laboratory-scale bioreactor experiments in this study suggest that comammox *Nitrospira* thrive under copper-limited conditions, while AOB are the predominant ammonia oxidizers when copper is replete, corroborating that copper availability is a key factor in the niche adaptation and competition between comammox *Nitrospira* and AOB. Additionally, even in the low-dCu bioreactor, nitrite oxidation was at least partly driven by canonical *Nitrospira*, which, like in the high-dCu bioreactor, even outnumbered the respective ammonia oxidizers in the system. This insight suggests that copper availability could be a potentially important parameter for influencing ammonia-oxidizing community composition in engineered systems.

### *In-situ* detection of comammox *Nitrospira amoA*

The use of 16S rRNA-targeted FISH probes is a frequent means to specifically detect *Nitrospira* in environmental samples, or to follow their enrichment in culture. The probe Ntspa476 was designed for the detection of the sequence cluster containing *Ca.* N. nitrosa and *Ca.* N. nitrificans [5]. However, while it is targeting a subset of lineage II *Nitrospira* that includes most known comammox *Nitrospira*, it also targets some sequences clustering with canonical *Nitrospira* [13]. Therefore, its use to reliably identify comammox *Nitrospira* is limited without prior confirmation that no canonical lineage II *Nitrospira* are present, which requires metagenomic sequencing combined with phylogenomic analyses [70]. Additionally, FISH probes targeting the 16S rRNA do not provide functional insight for the target organism. To address these limitations and enable the reliable detection of comammox *Nitrospira* in mixed communities, a function-specific in situ detection method for ammonia oxidation is required. Although mRNA-targeted CARD-FISH methods have been developed [71], they have not been applied to comammox *Nitrospira*.

**Figure 6:**
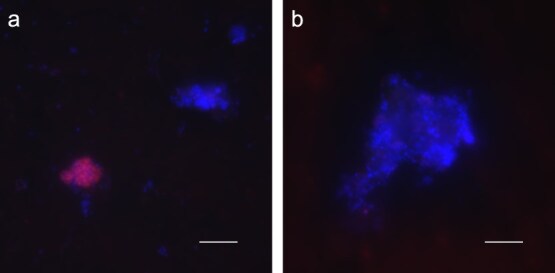
*AmoA* mRNA-targeted in situ detection of comammox *Nitrospira*. Biomass from the laboratory-scale bioreactors was harvested on day 180 from the (a) low-dCu and (b) high-dCu system. Comammox *Nitrospira* were stained by CARD-FISH with the probe Ntsp_amoA_249 (red) and all cells by DAPI (blue), resulting in magenta staining for comammox *Nitrospira*. The scale bars represent 10 μm.

We employed CARD-FISH with three newly designed probes targeting the comammox *Nitrospira amoA* mRNA for the specific detection and quantification of comammox *Nitrospira* in the bioreactor biomass. Initially, the HRP-labeled probes were tested against *E. coli* DH5α as a negative control to determine the optimal formamide (FA) concentration that minimized nonspecific probe binding and background fluorescence (Supplementary Fig. 4). In *E. coli*, the probes Ntsp_amoA_003 and Ntsp_amoA_642 yielded maximum signal intensities up to 40% FA, with intensities decreasing to background levels at 60% FA. Ntsp_amoA_249 showed maximal signal intensity up to 30% FA and remained slightly above background at 45% (Supplementary Fig. 4). Based on these results, we performed *amoA* mRNA-targeted CARD-FISH on the bioreactor biomass using probe Ntsp_amoA_249 at 45% FA. Quantification of probe-derived signals relative to DAPI staining showed that comammox *Nitrospira* constituted 12.0 ± 5.3% of the microbial community in the biomass from the low-dCu bioreactor on day 180 and was undetectable in the high-dCu bioreactor (Fig. 6, Supplementary Fig. 5, and Supplementary Table 6). This estimate closely matched the relative abundance determined by 16S rRNA gene amplicon analysis (11.8%; Fig. 5), indicating that the *amoA* mRNA-targeted CARD-FISH approach provides relative abundances comparable to those derived from 16S rRNA gene amplicon analysis in the studied system. Although this CARD-FISH approach was not designed to directly assess copper effects, the detection of comammox *Nitrospira* transcripts under low-dCu conditions supports their active role in ammonia oxidation in copper-limited environments, consistent with the observed community shifts.

In this study, the *amoA* gene sequences were identified in advance through metagenomic analysis, allowing the design and application of CARD-FISH for transcript detection. Recently, another powerful tool has been developed that directly links the ammonia-oxidizing function to phylogenetic identification [72]. This activity-based protein profiling method directly labels active AMO enzymes, thus potentially enabling the identification of novel ammonia-oxidizing microorganisms without requiring prior knowledge of functional gene sequences. In contrast, the CARD-FISH approach applied here enables the targeted detection and quantification of known ammonia-oxidizing microorganisms at the transcriptional level. These approaches are therefore complementary, with CARD-FISH providing high specificity for known populations and activity-based methods offering broader discovery potential.

## Conclusions

We conducted genome-centric analyses and laboratory-scale bioreactor experiments to investigate ecological differences between AOB and comammox *Nitrospira* in response to copper availability. Our findings suggest that comammox *Nitrospira* are well-adapted to a range of copper availabilities by encoding both low and high-affinity copper transport systems, allowing them to outcompete AOB at low copper concentrations, which only encode low-affinity copper transporters. Laboratory-scale bioreactor experiments confirmed that comammox *Nitrospira* were the dominant ammonia oxidizers in low-copper conditions, consistent with the genomic predictions. Furthermore, CARD-FISH targeting *amoA* mRNA confirmed the transcriptional activity of comammox *Nitrospira* under low-copper conditions, supporting their active role in ammonia oxidation. Together, these results provide new insights into the ecophysiology and niche adaptation of comammox *Nitrospira*. Considering such ecological differentiation among nitrifiers may provide valuable guidance for optimizing engineered nitrogen removal systems.

## Supplementary Material

Supplementary_material_ycag135

## Data Availability

The metagenomic sequencing data and the sequences of nitrifier MAGs from the full-scale bioreactor, as well as 16S rRNA gene amplicon sequencing data from the laboratory-scale bioreactors, have been deposited in the DNA Data Bank of Japan (DDBJ) under BioProject accession numbers PRJDB40319 and PRJDB40335, respectively.
