# Supplementary material for: Copper availability controls niche differentiation between comammox Nitrospira and ammonia-oxidizing bacteria
Source: ISME Commun. 2026 May 17;6(1):ycag135. doi: 10.1093/ismeco/ycag135 (PMC13374858; doi:10.1093/ismeco/ycag135)
Supplement: Supplementary_material_ycag135 [file supplementary_material_ycag135.zip › Kazuyoshi_2026_ISME_Comm_Supplementary_Information_ycag135.docx]

**Supplementary Information**

**Copper availability controls niche differentiation between comammox *Nitrospira* and ammonia-oxidizing bacteria**

Kazuyoshi Koike^1^, Garrett J. Smith^2,3^, Nao Okuda^1^, Ryoma Konno^4^, Shizuka Watanabe^4^, Yoshihito Kusunoki^4^, Shuji Kawakami^5^, Theo A. van Alen^2^, Maartje A.H.J. van Kessel^2^, Ryoko Yamamoto-Ikemoto^4^, Sebastian Lücker^2^, Norihisa Matsuura^4^#

^1^Graduate School of Natural Science and Technology, Kanazawa University, Kakuma, Kanazawa, 920-1192, Japan

^2^Department of Microbiology, Radboud Institute for Biological and Environmental Sciences, Radboud University, Nijmegen, The Netherlands

^3^Center of Microbiome Science, The Ohio State University, Columbus, Ohio 43210, USA

^4^Faculty of Geosciences and Civil Engineering, Kanazawa University, Kakuma, Kanazawa, 920-1192, Japan

^5^Department of Civil and Environmental Engineering, National Institute of Technology, Nagaoka College, 888, Nishi-Katagai, Nagaoka, 940-8531, Japan

# Corresponding author: Norihisa Matsuura (Kanazawa University)

Tel: +81-76-234-4640, Fax: +81-76-234-4640

matsuura@se.kanazawa-u.ac.jp

This supporting information contains 18 pages and 5 figures.

**1. Supplemental Methods**

**1.1 Sample collection and DNA extraction**

To collect biomass samples, 100 mL of 5 mm square sponge carriers from the full-scale bioreactor were collected at days 96, 231, 306, 393, 404, and 602 of operation, and squeezed to extract biomass [18]. An equal volume of 100% ethanol was added to preserve DNA in the samples. Before DNA extraction, the biomass was pelleted and washed twice with 1× phosphate-buffered saline (10,000 × g, 5 min, 4°C). DNA was extracted from 0.20 g of the washed pellet using the FastDNA SPIN Kit for Soil (MP Biomedicals, USA) following the manufacturer's instructions and stored at -20°C. The initial sample homogenization was performed twice at 4,500 rpm for 20 s with a Micro Smash MS-100 beads cell disruptor (TOMY, Japan), with subsequent recovery of the supernatant by centrifugation (14,000 × g, 5 min, 21°C).

**1.2 Illumina library preparation and sequencing**

For Illumina library preparation, the Nextera XT DNA Library Preparation Kit (Illumina, San Diego, CA, USA) was used according to the manufacturer's instructions. At least 1 ng of DNA per sample was enzymatically fragmented and tagmented, followed by the incorporation of indexed adapter sequences and amplification. Libraries were purified using AMPure XP beads (Beckman Coulter, Indianapolis, IN, USA) and checked for size distribution with the 2100 Bioanalyzer using the High Sensitivity DNA kit (Agilent, Santa Clara, CA, USA). Libraries were quantified with Qubit using the dsDNA HS Assay Kit (Thermo Fisher Scientific, Waltham, MA, USA). Finally, libraries were pooled at equimolar concentrations, denatured, and paired-end sequenced on an Illumina MiSeq or HiSeq X instrument. MiSeq Reagent Kit v3 (2 × 300 base pairs; Illumina) or v2 (2 × 250 base pairs; Illumina) was used for MiSeq sequencing, and HiSeq X Ten Reagent Kit v2.5 (2 × 150 base pairs; Illumina) was used for HiSeq X sequencing.

**1.3 Nanopore library preparation and sequencing**

For Nanopore library preparation, the Ligation Sequencing Kit 1D (SQK-LSK108) was used in combination with the Native Barcoding Expansion Kit (EXP-NBD104) according to the manufacturer’s protocol (Oxford Nanopore Technologies, Oxford, UK). At least 1 µg of DNA fragments per sample was damage-repaired using NEBNext FFPE DNA Repair Mix (New England Biolabs, Ipswich, MA, USA), end-repaired and dA-tailed using NEBNext Ultra II End Repair/dA-Tailing Module (New England Biolabs), and barcoded using the Blunt/TA Ligase Master Mix (New England Biolabs). After each treatment, the fragments were purified using AMPure XP beads. The DNA concentrations of all libraries were quantified with Qubit using the dsDNA HS Assay Kit and pooled. Subsequently, adapters were ligated to the DNA libraries using the NEBNext Quick Ligation Module (New England Biolabs), purified using AMPure XP beads, and pooled. Libraries were quantified again before being loaded into Flow Cell (R9.4.1) and sequenced on a MinION or GridION device (Oxford Nanopore Technologies, Oxford, UK) according to the manufacturer's instructions. Base calling was done using Guppy v4.0.11 with the dna_r9.4.1_450bps_hac.cfg model (Oxford Nanopore Technologies).

**1.4 Hybrid assembly approaches**

Sequencing read processing, hybrid assembly, and binning were performed following recent methods and recommendations, primarily that raw ONT assemblies tangibly benefit most for community reconstruction from long read correction and short read polishing between 2 and 5 iterations of each [1]. Raw Illumina paired-end sequencing reads (MiSeq and HiSeq) were processed by quality trimming, length filtering, adapter trimming, and contaminant filtering using BBDuk (BBTools v37.76; https://sourceforge.net/projects/bbmap/) by running bbduk.sh (settings qtrim=rl ktrim=r ref=adapters, artifacts, phix, lambda, mtst trimq=18 minlength=160 for MiSeq reads, or trimq=20 minlength=100 for HiSeq reads). Nanopore raw reads were filtered to a minimum read length of 3000 bp using BBMap (BBTools) by running the filterbyname.sh command (minlen=3000). Porechop v0.2.4 (https://github.com/rrwick/Porechop) was used with default parameters to detect and split chimeras containing the adapter in the middle of a read. If the resulting fragments were shorter than 3000 bp, they were discarded with the option --min_split_read_size 3000. These quality-controlled reads will be referred to as QC Illumina or QC Nanopore reads. The QC reads were then hybrid-assembled as described below with both (i) long-read-first assembly and correction for time points with sufficient data in QC Nanopore reads, using Illumina reads to correct errors in the assembled and corrected contigs, or (ii) short-read-first assembly for time points with insufficient data in QC Nanopore reads, using Nanopore reads to bridge assembled contigs and scaffold them.

(i) The QC Nanopore reads were *de novo* assembled using Flye v2.9 9 [2] with parameters --nano-raw and --meta. Nanopore assemblies were statistically evaluated using BBMap (BBTools v37.76; <https://sourceforge.net/projects/bbmap/>) and SeqKit v0.7.1 [3] with the bbstats.sh and stats commands, respectively. Contigs were then separated into circular and non-circular using the output information of Flye via the command line and BBMap using the utility filterbyname.sh, and processed separately as all non-circular contigs and all circular contigs. For self-error correction of non-circular contigs, QC Nanopore reads were mapped to long-read assemblies using Minimap2 v2.16-r922 [4] with the setting -c -x map-ont and were then error corrected using Racon v1.3.1 (https://github.com/lbcb-sci/racon) with the option --include-unpolished to retain contigs that were not corrected. This was repeated using the newly corrected contigs as the reference. Next, the error-corrected contigs were polished using QC Illumina data by mapping the reads using the bbmap.sh command in BBMap, and subsequent polishing using Pilon v1.23 [5]. In total, 10 rounds of short-read polishing were performed, as it was apparent that some changes were still made after 5 iterations. Circular contigs were polished five times using the same process but without preceding long-read error correction because the need to correct larger-scale mis-assemblies, e.g., inversions, for which Nanopore reads are suited, is minimal for a possibly complete genome compared to the smaller-scale mis-assemblies, e.g., insertions, deletions, and substitutions, for which use of Nanopore reads are less well-suited when short reads are available. For each round of short-read polishing, protein-coding genes on the polished contigs were predicted by Prodigal v2.6.3 [6] with the setting -p meta. Then, the theoretical contamination level of the assembly was assessed using the taxonomy_wf of CheckM v1.1.3 [7] with the setting --genes. As the CheckM contamination (redundancy of markers) estimate reflects the number of duplicated marker genes recovered, it can be expected that this number will increase when frameshifts typical of Nanopore long-read sequencing are fixed by short-read polishing, resulting in a larger number of marker proteins identified [1]. The polishing round with the largest apparent contamination, in this case as a proxy for community reconstruction capability, was then selected for downstream analysis, which was reached after the seventh polishing round for the non-circular contigs and after the fifth polishing round for the circular contig.

(ii) The QC Illumina reads were *de novo* hybrid-assembled using metaSPAdes v3.14.0 [8], adding the QC Nanopore reads with the parameter --nanopore. Here, the long reads are used for gap closure and repeat resolution. The resulting hybrid assemblies were evaluated using the bbstats.sh command of BBMap.

**1.5 Composition of the culture medium supplied to the laboratory-scale bioreactor**

The cultivation medium consisted of (L^‑1^): 44.0 mg (NH_4_)_2_SO_4_, 10.0 mg CaCO_3_, 87.7 mg NaCl, 50.0 mg MgSO_4_×7H_2_O, 150 mg KH_2_PO_4_, 1 mL of modified trace element solution (TES), and 1 mL of selenium-wolfram solution (SWS) [9]. The two modified TES with different dissolved copper (dCu) concentrations contained (L^-1^): 49.1 mg MnSO_4_×5H_2_O, 50 mg H_3_BO_3_, 70 mg ZnCl_2_, 72.6 mg Na_2_MoO_4_×2H_2_O, 24 mg NiCl_2_×6H_2_O, 80 mg CoCl_2_×6H_2_O, 1 g FeSO_4_×7H_2_O, and 0.54 or 537 mg CuCl_2_×2H_2_O for the low-dCu and high-dCu conditions, respectively. All salts besides FeSO_4_×7H_2_O were dissolved in Milli-Q water. Subsequently, 2.5 ml of 37 % HCl was added before dissolving the FeSO_4_×7H_2_O. The SWS contained (L^-1^): 0.5 g NaOH, 3 mg Na_2_SeO_3_×5H_2_O, and 4 mg Na_2_WO_4_×2H_2_O. The medium was adjusted to pH 7.8 with 1 M KHCO_3_ and purged with nitrogen gas to reduce oxygen levels.

**1.6 16S rRNA gene amplicon sequencing**

To study the dynamics within the nitrifying microbial communities, sponges were collected from each bioreactor at 0 (inoculum sponges from a full-scale bioreactor), 15, 35, 50, 70, 90, 120, 150, and 180 days. Biomass was extracted by squeezing four sponges (totaling 0.5 mL) into 2.0 mL tubes containing 0.5 mL of 1× phosphate-buffered saline (PBS). 0.25 mL of the obtained microbial cell suspension was immediately transferred to a Lysing Matrix E tube for DNA extraction using the FastDNA SPIN Kit for Soil. The DNA was stored at -20°C for subsequent analysis.

For 16S rRNA gene amplicon sequencing, libraries were prepared using a two-step PCR protocol. The first-round PCR reactions (30 µL), consisting of a mix of 1× PCR buffer (Qiagen, Hilden, Germany), 250 µM of each deoxynucleotide triphosphate (dNTP Mixture; Takara Bio, Kusatsu, Japan), 200 nM each primer, 0.5 units of HotStarTaq Plus DNA Polymerase (Qiagen), and 3 ng µL^-1^ of DNA template. The used primers were 515F (5’- GTGCCAGCMGCCGCGGTAA -3’) and 806R (5’- GGACTACHVGGGTWTCTAAT -3’) [10], which were modified to include Illumina adapter sequences. The thermal cycling included an initial heat activation at 95 °C for 5 minutes, followed by 27 cycles of amplification at 94 °C for 30 sec, 55 °C for 30 sec, and 72 °C for 1 min, and a final elongation step at 72 °C for 10 minutes. The resulting amplicons were purified using the Agencourt AMPure XP system by adding 1.0 volumes of bead solution.

The second-round PCR (25 µL) used 1× KAPA HiFi HotStart ReadyMix (Kapa Biosystems, Wilmington, Massachusetts, USA), 300 nM each of the Nextera XT index adapters (Illumina), and 2 ng µL^-1^ of purified first-round PCR product. Thermocycling was performed as follows: 95 °C for 3 min; 10 cycles of 98 °C for 20 sec, 60 °C for 15 sec, and 72 °C for 1 min; and 72 °C for 1 min. Second-round PCR products were purified with 1.0 volumes of AMPure XP beads and quantified using the DNA 1000 assay system with a 2100 Bioanalyzer instrument (Agilent). The amplicons were then pooled at equimolar concentrations and sequenced on an Illumina MiSeq platform using the MiSeq Reagent Nano Kit v2 (2 × 250 bp; Illumina). A PhiX Control v3 (Illumina) was added to the library at a concentration of 30 % (vol/vol).

The raw sequencing data were processed by trimming and filtering of the reads using BBDuk with the bbduk.sh command (setting qtrim=rl trimq=18 minlength=200), followed by Cutadapt v4.1 [11] to remove primer sequences allowing up to two mismatches (-e 0.12) and discarding reads without adapter (--discard-untrimmed). Using DADA2 v1.30.0 [12], each dataset was filtered and trimmed (maxEE=c(2,2), truncLen=c(200,200)), followed by error rate learning (nbases=1e10), dereplication of the reads (default parameters), Amplicon Sequence Variant (ASV) inference (pool=TRUE), merging of the paired reads (default parameters), and chimera removal (default parameters). Finally, ASVs were taxonomically classified using the SILVA 138.1 prokaryotic SSU taxonomic training data formatted for DADA2 [13].

**1.7 Preliminary laboratory-scale bioreactor operation and DNA sequencing**

Prior to the two laboratory-scale bioreactors operated at different copper concentrations described in the main text, we conducted three preliminary laboratory-scale bioreactor experiments (pre_01, pre_02, and pre_03) to investigate nitrifier dynamics under controlled copper conditions. The reactors used were identical to those described in the main manuscript (Fig. 1), and the media were prepared in the same manner as described above. The preliminary experiments differed in copper conditions, influent flow rates, and inoculation strategies. Experiments pre_01 and pre_02 were operated only under low-dCu, whereas experiment pre_03 included both low-dCu and high-dCu reactor series. In experiment pre_01, the flow rate was adjusted stepwise during operation: 50 mL h^-1^ from day 0 to 20, 80 mL h^-1^ from day 21 to 45, 100 mL h^-1^ from day 46 to 75, and 200 mL h^-1^ from day 76 to 90. In contrast, the flow rates in experiments pre_02 and pre_03 were maintained at 100 mL h^-1^ throughout the entire operational period. Inoculums collected at different three time points from the full-scale bioreactor were used for each preliminary experiment. In experiments pre_01 and pre_02, sponge carriers containing biomass from the inoculum source were directly transferred into the preliminary laboratory-scale bioreactor. In contrast, in experiment pre_03, biomass was squeezed from the inoculum sponge and used as the inoculum and supplemented with fresh sponge carriers as described in the main text. For DNA sequencing, biomass samples were collected at multiple time points during each preliminary experiment. In experiment pre_01, biomass was sampled on days 0, 20, 45, 75, and 90. In experiment pre_02, biomass was sampled on days 0, 15, 70, 90, 120, 150, and 180. In experiment pre_03, biomass was collected from both the low-dCu and high-dCu reactors on days 0, 15, and 30. For all samples, biomass collection, DNA extraction, PCR amplification, 16S rRNA gene amplicon sequencing, and downstream data processing were performed following the same procedures described in the main text.

**1.8 Phylogenetic analysis based on 16S rRNA gene**

For phylogenetic analysis of the 16S rRNA gene amplicons from the laboratory-scale bioreactor, reference sequences were obtained from NCBI (March 18, 2022) and filtered at >1000 bp using SeqKit. Our *Nitrospira* ASV sequences (253 bp) were aligned with the reference gene sequences using MAFFT v7.215 [14]. The phylogenetic tree was calculated using IQ-TREE v2.1.3 [15] with the best-fit model (-m MFP) and 1,000 ultrafast bootstrapping replications (-B 1000). Final trees were visualized in iTOL v6.6 [16].

**1.9 Comammox *amoA* gene-specific probe design**

To specifically detect comammox *Nitrospira* in the laboratory-scale bioreactors, a probe targeting the comammox *amoA* mRNA was developed. First, 16S rRNA gene sequences were extracted using Barrnap v0.9 from comammox *Nitrospira* MAGs obtained from a full-scale bioreactor metagenome and aligned with the *Nitrospira* ASV sequence (253 bp) identified in the laboratory-scale bioreactors to identify the closest relative. For the MAG that was a perfect match to the dominant *Nitrospira* ASVs (SFBR_MAG_41), we aimed to design an *amoA*-targeted oligonucleotide probe that could specifically detect this comammox *Nitrospira* and its closest relatives.

We identified a total of 826 probe candidates by moving an 18 bp sliding window across the 843 bp comammox *amoA* gene of this *Nitrospira* MAG one base at a time. Subsequently, we screened all candidates for matches to additional comammox *amoA* sequences. To build the reference dataset, we obtained all accession numbers of members of the family *Nitrospiraceae* registered in the GTDB r207 (194 genomes) and downloaded the corresponding assemblies from the NCBI FTP server (<https://www.ncbi.nlm.nih.gov/>; January 31, 2023) using ncbi-genome-download v0.3.1 (https://github.com/kblin/ncbi-genome-download) with options --section refseq and --section genbank. Protein-coding sequences in the genome assemblies were predicted using Prodigal with setting -p meta, and the translated amino acid sequences of the *amoA* genes were identified using hmmlearn v0.2.8 (<https://github.com/hmmlearn/hmmlearn>) with PF02461 from the Pfam 35.0 database [17]. To reduce false positives, extracted genes were filtered against an in-house database downloaded from TrEMBL and Swiss-Prot (February 1, 2023) using BLAST with option -evalue 1e-6, -qcov_hsp_perc 50, and manually filtered at 60 % amino acid identity. This procedure yielded a curated *amoA* reference database comprising 67 sequences within the family *Nitrospiraceae*. For this curated database, we determined the counts of 0, 1, 2, and 3 mismatches to the 826 probe candidates using Cutadapt v4.1 with settings --overlap 18 --error-rate 0, 0.08, 0.13, and 0.2, respectively. The top two sequences with maximal counts for 0 and 1 mismatches were selected (Ntsp_amoA_003 [5’− ATTTCATCGGTTCTAAAC −3’] and Ntsp_amoA_642 [5’− GAGATCATGGTGCTGTGA −3’]). Fourth, we confirmed that the probe candidates did not match any non-comammox *Nitrospira* gene sequences within the PmoA/AmoA protein family, including *amoA* sequences of the families *Nitrosomonadaceae* and *Nitrosococcaceae* and the order *Nitrososphaerales*, and *pmoA* sequences of the families *Beijerinckiaceae*, *Methylacidiphilaceae*, *Methylococcaceae*, *Methylomirabilaceae*, and *Methylothermaceae*. These gene sequences were extracted as described above, except that the PF12942 HMM was used to identify the archaeal AmoA. Additionally, we selected one specific probe (Ntsp_amoA_249 [5’− GCTGACGATAGTTCACCCAA −3’]) targeting our previously reported partial comammox *amoA* gene sequence (coma *amoA* ASV2, 158 bp) [18]. The numbers of mismatches between these three probes and the 67-reference *amoA* sequences are summarized (Supplementary Table 2). The three probes were synthesized containing a horseradish peroxidase (HRP) modification at the 5' end (Japan Bio Services, Saitama, Japan).

**1.10 CARD-FISH-based detection of comammox *Nitrospira***

CARD-FISH was performed as follows: 4 µL of sample in PBS/ethanol was mixed with 2.4 µL of 1× PBS and 0.8 µL of 0.001 % sodium dodecyl sulfate (SDS), and embedded in 0.8 µL of 0.1 % low-melting-point agarose (Agarose XP; NIPPON GENE, Tokyo, Japan) at 60 °C. The samples were dried at 60 °C for 10 min and dehydrated for 3 min each with 50, 80, and 99.5 % ethanol. For permeabilization of bacterial cell walls, lysozyme solution (10 mg/mL of lysozyme, 50 mM EDTA, and 0.1 M Tris-HCl) was pipetted onto the wells (20 µL/well), and slides were incubated in a humid chamber containing tissue paper soaked in MilliQ at 37 °C for 60 min. After lysozyme treatment, the slides were immersed in 0.05 % Triton X-100 in 1× PBS for 10 min, MilliQ water for 1 min, dehydrated in 99.5 % ethanol for 1 min, and finally air-dried.

For *in situ* hybridization, each sample on the slide was covered with 14 µL of hybridization buffer (0.9 M NaCl, 20 mM Tris-HCl [pH 8.0], 10 % sodium dextran sulfate [w/v], 0.01 % SDS [w/v], 10-60 % formamide [v/v], Supplementary Table 7) containing 0.25 mM HRP-labeled probe and incubated at 46 °C for 5 h in a 50-mL tube containing a humidified tissue paper soaked with hybridization buffer (without probe), followed by washing in pre-warmed washing buffer (14-900 mM NaCl, 20 mM Tris-HCl [pH 8.0], 5 mM EDTA [pH 8.0], 0.01 % SDS [w/v]) at 48 °C for 20 min. Before tyramide signal amplification (TSA), the probe-bound HRP was equilibrated by incubating with 0.05 % Triton X-100 in 1× PBS for 15 min at room temperature. After careful removal of excess buffer, each well on the slide was covered with 10 µL of TSA reaction buffer containing 0.98× Plus Amplification Diluent (Akoya Biosciences, Marlborough, MA, USA), 0.02× TSA Plus Cy3 Regent dissolved in 150 µL dimethyl sulfoxide (Akoya Biosciences, Marlborough, MA, USA), and incubated in a humidified chamber (with MilliQ) at 37 °C for 15 min. After signal amplification, slides were washed with 1× PBS supplemented with 0.05 % Triton X-100 for 15 min at room temperature, and with MilliQ water for 1 min. The slides were dehydrated in 99.5 % ethanol for 1 min and air-dried. Finally, SlowFade Diamond Antifade Mountant with DAPI (Thermo Fisher Scientific, Waltham, MA, USA) was dropped on each well, and microscopy was performed as described below. As a negative control sample, *Escherichia coli* DH5α (NIPPON GENE, Tokyo, Japan) was cultured (LB medium, 37 °C, overnight), washed once with 1× PBS with centrifugation at 8000 × *g* for 10 min, and PFA fixed. CARD-FISH was then performed as described above. In addition, a negative control without probes was performed using *E. coli* DH5α and biomass samples with the method described above, but without the addition of probes.

The fluorescence of cells was detected on an ECLIPSE Ni-U microscope (Nikon, Tokyo, Japan) equipped with a halogen and LED light source (four types of main wavelengths of 385, 475, 550, and 621 nm; D-LEDI-C, Nikon) and filter sets for detecting DAPI, FITC, and Cy3 (Nikon). Fluorescence images were recorded in grayscale with a 100× oil immersion objective (Plan Fluor; Nikon) at a resolution of 6000 × 3984 pixels and 8-bit depth using a Digital Sight 10 digital camera (Nikon) and NIS-Elements BR v5.41.00 software (Nikon). All CARD-FISH experiments were conducted in technical duplicates.

**2. Supplemental Results**

**2.1 Nitrifier dynamics in preliminary laboratory-scale bioreactors**

We operated three independent preliminary laboratory-scale bioreactors. In all reactors, complete ammonium removal was achieved within five days after startup, and stable nitrification performance was maintained thereafter (data not shown). Nitrifier community dynamics in each bioreactor were investigated based on relative abundances derived from 16S rRNA gene amplicon sequencing. In all three bioreactors operated under low-dCu (0.2 µg L^-1^) conditions, comammox *Nitrospira* became more abundant than AOB by the end of operation. In contrast, in the single bioreactor operated under high-dCu (200 µg L^-1^) conditions, AOB dominated over comammox *Nitrospira* at the final sampling time (Supplementary Figure 3). Notably, in experiment pre_01, comammox Nitrospira, which were initially approximately 15-fold less abundant than AOB in the inoculum, became dominant over AOB by the end of the operational period. The ASV table derived from 16S rRNA gene sequencing of all laboratory-scale bioreactors in this study is available in Supplementary Table 8.

**Supplementary Figure 1: Carbon fixation pathways and nitrogen-cycling enzyme complexes conserved in the nitrifier MAGs.** The heat map indicates the completeness of pathways and protein complexes. Canonical nitrite-oxidizing bacteria are highlighted in green, complete ammonia-oxidizing bacteria in red, and ammonia-oxidizing bacteria in blue.

**Supplementary Figure 2: 16S rRNA gene-based phylogenetic tree showing the affiliation of *Nitrospira* sequences obtained from the full-scale and laboratory-scale bioreactors.** The maximum-likelihood tree was constructed with the same sequences as in Fig. 2A, plus the 16S rRNA gene amplicon ASVs obtained from the laboratory-scale bioreactor. MAG sequences obtained in this study are indicated in red, ASVs in green, and characterized comammox *Nitrospira* in blue. Support values from 1,000 ultrafast bootstrapping replications are represented by brown circles. The scale bar corresponds to 10 % sequence divergence.

**Supplementary Figure 3: Nitrifier dynamics based on the 16S rRNA gene amplicon sequencing in the preliminary laboratory-scale bioreactors. (A)** Relative abundance of nitrifiers under the low-dCu stepwise adjusted flow rate condition (pre_01). **(B)** Relative abundance of nitrifiers under the low-dCu condition (pre_02). **(C)** Relative abundance of nitrifiers under the low-dCu and high-dCu conditions (pre_03). Unless otherwise stated, the bioreactors were operated at a flow rate of 100 mL h^-1^.

**Supplementary Figure 4: Signal intensities of the three *amoA* mRNA-targeting CARD-FISH probes at increasing formamide (FA) concentrations using *E. coli*.** The data points represent the mean of 4 to 20 technical replicates, and the error bars represent their standard deviation. The background fluorescence (no-probe control) was measured only at 10 % FA.

**Supplementary Figure 5: Representative micrographs of comammox *Nitrospira* in biomass collected from the laboratory-scale bioreactors at day 180.** **(A)** Gray-scale image of the biomass stained with DAPI. **(B)** Gray-scale image of the *amoA* mRNA probe-derived fluorescent signal (Cy3) specific for comammox *Nitrospira*. **(C)** Overlay of the DAPI (blue) and Cy3 (red) channels showing comammox *Nitrospira* in magenta. Images from a hybridization without probes are shown as a negative control. Scale bars in all images correspond to 10 µm.

**Reference**

1. Smith GJ, van Alen TA, van Kessel MAHJ, Lücker S. Simple, reference-independent assessment to empirically guide correction and polishing of hybrid microbial community metagenomic assembly. *PeerJ* 2024;12:e18132. https://doi.org/10.7717/peerj.18132

2. Kolmogorov M, Yuan J, Lin Y, Pevzner PA. Assembly of long, error-prone reads using repeat graphs. *Nature Biotechnol* 2019;37:540–546. https://doi.org/10.1038/s41587-019-0072-8

3. Shen W, Le S, Li Y, Hu F. SeqKit: A Cross-Platform and Ultrafast Toolkit for FASTA/Q File Manipulation. *PLoS One* 2016;11:e0163962. https://doi.org/10.1371/journal.pone.0163962

4. Li H. Minimap2: pairwise alignment for nucleotide sequences. *Bioinformatics* 2018;34:3094–3100. https://doi.org/10.1093/bioinformatics/bty191

5. Walker BJ, Abeel T, Shea T, Priest M, Abouelliel A, Sakthikumar S et al. Pilon: an integrated tool for comprehensive microbial variant detection and genome assembly improvement. *PLoS One* 2014;9:e112963. https://doi.org/10.1371/journal.pone.0112963

6. Hyatt D, Chen GL, Locascio PF, Land ML, Larimer FW, Hauser LJ. Prodigal: prokaryotic gene recognition and translation initiation site identification. *BMC Bioinformatics* 2010;11:119. https://doi.org/10.1186/1471-2105-11-119

7. Parks DH, Imelfort M, Skennerton CT, Hugenholtz P, Tyson GW. CheckM: assessing the quality of microbial genomes recovered from isolates, single cells, and metagenomes. *Genome Res* 2015;25:1043–1055. https://doi.org/10.1101/gr.186072.114

8. Nurk S, Meleshko D, Korobeynikov A, Pevzner PA. metaSPAdes: a new versatile metagenomic assembler. *Genome Res* 2017;27:824–834. https://doi.org/10.1101/gr.213959.116

9. Daims H, Lebedeva EV, Pjevac P, Han P, Herbold C, Albertsen M et al. Complete nitrification by *Nitrospira* bacteria. *Nature* 2015;528:504–509. https://doi.org/10.1038/nature16461

10. Caporaso JG, Lauber CL, Walters WA, Berg-Lyons D, Huntley J, Fierer N et al. Ultra-high-throughput microbial community analysis on the Illumina HiSeq and MiSeq platforms. *ISME J* 2012;6:1621–1624. https://doi.org/10.1038/ISMEJ.2012.8

11. Martin M. Cutadapt removes adapter sequences from high-throughput sequencing reads. *EMBnet J* 2011;17:10. https://doi.org/10.14806/ej.17.1.200

12. Callahan BJ, McMurdie PJ, Rosen MJ, Han AW, Johnson AJ, Holmes SP. DADA2: High-resolution sample inference from Illumina amplicon data. *Nat Methods* 2016;13:581–583. https://doi.org/10.1038/nmeth.3869

13. Quast C, Pruesse E, Yilmaz P, Gerken J, Schweer T, Yarza P et al. The SILVA ribosomal RNA gene database project: improved data processing and web-based tools. *Nucleic Acids Res* 2013;41:D590–D596. https://doi.org/10.1093/nar/gks1219

14. Katoh K, Misawa K, Kuma K, Miyata T. MAFFT: a novel method for rapid multiple sequence alignment based on fast Fourier transform. *Nucleic Acids Res* 2002;30:3059–3066. https://doi.org/10.1093/nar/gkf436

15. Minh BQ, Schmidt HA, Chernomor O, Schrempf D, Woodhams MD, von Haeseler A et al. IQ-TREE 2: New Models and Efficient Methods for Phylogenetic Inference in the Genomic Era. *Mol Biol Evol* 2020;37:1530–1534. https://doi.org/10.1093/molbev/msaa015

16. Letunic I, Bork P. Interactive Tree of Life (iTOL) v6: recent updates to the phylogenetic tree display and annotation tool. *Nucleic Acids Res* 2024;52:W78–W82. https://doi.org/10.1093/nar/gkae268

17. Mistry J, Chuguransky S, Williams L, Qureshi M, Salazar GA, Sonnhammer ELL et al. Pfam: The protein families database in 2021. *Nucleic Acids Res* 2021;49:D412–D419. https://doi.org/10.1093/nar/gkaa913

18. Koike K, Smith GJ, Yamamoto-Ikemoto R, Lücker S, Matsuura N. Distinct comammox *Nitrospira* catalyze ammonia oxidation in a full-scale groundwater treatment bioreactor under copper limited conditions. *Water Res* 2022;210:117986. https://doi.org/10.1016/j.watres.2021.117986
